# LXR Activation Induces a Proinflammatory Trained Innate Immunity-Phenotype in Human Monocytes

**DOI:** 10.3389/fimmu.2020.00353

**Published:** 2020-03-10

**Authors:** Yahya Sohrabi, Glenn V. H. Sonntag, Laura C. Braun, Sina M. M. Lagache, Marie Liebmann, Luisa Klotz, Rinesh Godfrey, Florian Kahles, Johannes Waltenberger, Hannes M. Findeisen

**Affiliations:** ^1^Department of Cardiology I – Coronary and Peripheral Vascular Disease, Heart Failure, University Hospital Münster, Münster, Germany; ^2^Clinic of Neurology with Institute of Translational Neurology, University Hospital Münster, Münster, Germany; ^3^Department of Internal Medicine I–Cardiology, University Hospital Aachen, Aachen, Germany; ^4^Department of Cardiology and Angiology, University Hospital Münster, Münster, Germany; ^5^Cells-in-Motion Cluster of Excellence (EXC 1003 – CiM), University of Münster, Münster, Germany

**Keywords:** trained innate immunity, monocytes, LXR, acetyl-CoA, inflammation

## Abstract

**Objectives:**

The concept of trained innate immunity describes a long-term proinflammatory memory in innate immune cells. Trained innate immunity is regulated through reprogramming of cellular metabolic pathways including cholesterol and fatty acid synthesis. Here, we have analyzed the role of Liver X Receptor (LXR), a key regulator of cholesterol and fatty acid homeostasis, in trained innate immunity.

**Methods and Results:**

Human monocytes were isolated and incubated with different stimuli for 24 h, including LXR agonists, antagonists and Bacillus Calmette-Guerin (BCG) vaccine. After 5 days resting time, cells were restimulated with the TLR2-agonist Pam3cys. LXR activation did not only increase BCG trained immunity, but also induced a long-term inflammatory activation by itself. This inflammatory activation by LXR agonists was accompanied by characteristic features of trained innate immunity, such as activating histone marks on inflammatory gene promoters and metabolic reprogramming with increased lactate production and decreased oxygen consumption rate. Mechanistically, LXR priming increased cellular acetyl-CoA levels and was dependent on the activation of the mevalonate pathway and IL-1β signaling. In contrast to mevalonate pathway inhibition, blocking fatty acid synthesis further increased proinflammatory priming by LXR.

**Conclusion:**

We demonstrate that LXR activation induces a proinflammatory trained immunity phenotype in human monocytes through epigenetic and metabolic reprogramming. Our data reveal important novel aspects of LXR signaling in innate immunity.

## Introduction

Innate immune cells such as monocytes and macrophages retain a high phenotypic plasticity in response to environmental cues to be able to rapidly fulfill their diverse functions in host defense and tissue homeostasis (1). It has become increasingly apparent that a comprehensive rewiring of cellular metabolic pathways provides the foundation for this phenotypic flexibility. The term immunometabolism has been introduced to describe this regulation of immune cell function through intracellular metabolic pathways. Metabolic modulation of immune cells can rapidly satisfy increased energy demands through enhanced aerobic glycolysis, or provide metabolic intermediates that can be used for cell growth, signaling or production of inflammatory mediators (2, 3). In addition, metabolic reprogramming controls epigenetic modifications, implementing long-term phenotypic changes. Recently Netea et al. have introduced the concept of trained innate immunity or innate immune memory to describe a long-term adaption of innate immune cells to respond to certain stimuli with an enhanced inflammatory response (4). *In vitro* and *in vivo* treatment with the Bacillus Calmette-Guerin (BCG) vaccine or the cell wall components of Candida albicans (β-glucan) trained monocytes and macrophages to respond to TLR restimulation with increased synthesis of chemokines and cytokines, offering a survival benefit in models of infectious diseases (5, 6). Additionally, sterile inflammatory triggers such as oxidized low-density lipoprotein (oxLDL) can also induce a sustained activation of innate immune cells, potentially contributing to atherosclerosis and other chronic inflammatory diseases (7–11). Immunometabolic reprogramming of various pathways including glycolysis, oxidative phosphorylation, glutaminolysis as well as fatty acid and cholesterol metabolism controls trained innate immunity (5). Metabolic intermediates regulate specific epigenetic enzymes leading to the enrichment of activating histone marks on the promoters of inflammatory genes (12). Recent evidence has highlighted the crucial role of cholesterol and fatty acid synthesis in regulating trained innate immunity (10, 13). Training strongly induces several key enzymes of the cholesterol synthesis pathway, while inhibition of the rate limiting enzyme high-mobility group (HMG)-coenzyme A (CoA)-reductase with Fluvastatin blocked the emergence of a trained phenotype. Furthermore, fatty acid metabolites are involved in the regulation of trained-immunity-associated effects on hematopoietic progenitors *in vivo* (14).

Liver X receptors (LXRs) are ligand activated nuclear receptor transcription factors that constitute key cellular regulators of both cholesterol and fatty acid metabolism. LXR functions include sensing of intracellular cholesterol levels and the regulation of reverse cholesterol transport (RCT) through its target genes *ABCA1* and *ABCG1*. Furthermore, LXRs regulate expression of genes involved in fatty acid synthesis including *SREBP1c* and *FASN* (15). Additionally, there is evidence demonstrating anti-inflammatory effects of LXR activation in murine innate immune cells (16). Due to their metabolic and anti-inflammatory effects, LXR agonists have been suggested as novel therapeutic options for metabolic and inflammatory diseases such as atherosclerosis (17). However, the anti-inflammatory effects of LXR agonists in innate immune cells were observed primarily in murine macrophages. In contrast, preliminary experiments in human macrophages demonstrating proinflammatory effects of LXR agonists suggest a more complex role of LXR in human innate immunity (18). Considering the extensive evidence demonstrating regulatory functions of LXRs in innate immunity and lipid metabolism, we have studied the effect of LXR activation in monocyte trained innate immunity.

## Materials and Methods

### PBMC and Monocyte Isolation

Human monocytes were isolated from fresh human blood leukocyte reduction chambers of platelet apheresis sets from healthy subjects (non-smokers, 18–55 years old) recruited by the blood bank of the University Hospital Münster as described earlier (8). The study was approved by the scientific and ethics committee of the University of Münster and conforms to the principles of the Declaration of Helsinki. Written informed consent was obtained from all donors by the blood bank and leukocyte reduction filters were provided anonymously without sharing additional personal and detailed information. The general response to the applied treatments was observed in all samples.

Monocyte isolation was then performed by differential density centrifugation over Histopaque^®^ 1077 (Sigma, #10771) using Leucosep^®^ tubes (50 ml, with filter, Greiner, # 227290). The cells were washed in PBS until the cell suspension in a 50 ml falcon tube looked transparent. In order to enrich monocytes, a second density centrifugation using percoll gradient was performed. Briefly, 150–200 × 10^6^ PBMCs were resuspended in RPMI-1640 (Sigma, #R8758) with 10% FBS (Sigma, #F7524), layered on top of a hyper-osmotic Percoll (GE Healthcare, #17089101) solution (46% Percoll and PBS, 10%FBS RPMI) and centrifuged for 30 min at 580 *g*. The interphase layer was isolated and cells were washed with PBS. Cells were purified further with MACS Pan Monocyte Isolation Kit (Miltenyi Biotec, #130-096-537) and washed once with serum-free RPMI-1640 medium before resuspension in RPMI culture medium supplemented with 10% pooled AB human serum (Sigma, H4522), 1% penicillin/streptomycin (Gibco, # 15140122) and 5 mM glucose (Sigma, G8644).

### Monocyte Priming Experiments

Monocytes were primed by culturing 40.000 cells/well in a 96-well plate (*Greiner Bio-One^TM^*) with 2 μg/ml BCG, 2 μM T0901317 (T1317) (Cayman, #71810), 10 μM GW3965 (Cayman, #10054), 5 μM GSK2033 (Cayman, #25443), 500 μM acetyl-CoA (Cayman, # 16160) or 10 ng/ml IL-1β (Peprotech, #200-01A) for 24 h in RPMI supplemented with 10% pooled human AB serum, 5 mM glucose and 1% Penicillin/Streptomycin. In experiments in which inhibitors were used, cells were pre-incubated for at least an hour with 100 nm Torin1 (Cayman, #10997), 20 μM MTA (Cayman, #15593), 10 μM LW6 (Axonmedchem, # 2480), 10 μM KC7F2 (Tocris, #4324), 20 μM Fluvastatin (Sigma, SML0038), 25 μM C75 (Tocris, #2489), 1 μM diacerein (Cayman,# 11710), 200 ng/ml IL-1RA (Peprotech, #200-01RA) prior to priming. The medium was changed after 24 h and cells were rested for 5 days or as indicated. 50% of the medium was refreshed on day 3. On day 6 medium was changed and cells were re-stimulated with either 200 μL RPMI containing 5 μg/ml of Pam3Cys (EMC mirocollection, #L2000), 10 ng/ml of LPS (Sigma) or with medium only. DMSO control was always included when there was a compound dissolved in DMSO. All working solutions were prepared in culture media. The plates were incubated in a 5% CO2 incubator maintained at 37°C. After 24 h supernatants were collected and stored at −20°C until used for cytokine assay.

### Cytokine Measurements

The levels of the proinflammatory cytokines in supernatants were measured using DuoSet ELISA kits for human TNFα (R&D, #DY210) and human IL-6 (R&D, #DY206), IL-8 (R&D, #DY208), MCP-1 (R&D, #DY279) and IL-1β (R&D, #DY401) following the instructions of the manufacturer. The absorbance was quantified in a Multimode Plate Reader Victor X3, P Perkin Elmer (United States) at 450 nm. Concentrations were calculated by four parameters logistic regression.

### Gene Silencing by siRNA

Cells were seeded on either 12- or 96-well cell culture treated plates at a concentration of 1000,000 and 60,000 cells/well, respectively, and transfection experiment was performed as it was described earlier (8). The cells were transfected with final concentrations of 60 nM small interfering RNA for SREBP1 (Santa Cruz, # sc-36557) for 24 h using Viromer technology according to the manufacturer’s protocol (Viromer Green, Lipocalyx). Scramble siRNA (Santa Cruz, #sc-37007) was used as a control for the experiments. Knockdown of *SREBP1* was confirmed by qPCR analysis 24 h post transfection. For further analysis, 24 h after transfection cells were either left untreated or treated with 2 μM T1317 as described earlier in our monocyte priming protocol. IL-6 and TNFα concentrations were analyzed as described in the respective paragraphs of the section “Materials and Methods.”

### RNA Isolation and qPCR

For real-time qPCR trained monocytes were lysed after 24 h or on day 6 after priming for mRNA isolation. For analyzing expression of cytokines, the cells were stimulated with 5 μg/ml of Pam3Cys for 6 h before lysing. Total RNA purification was performed using NucleoSpin RNA-isolation kit (Macherey-Nagel) and reverse-transcribed using the RevertAid First Strand cDNA Synthesis Kit (Thermo Scientific). Expression of *AACS, ACSL1, ACSL3, ACSS2, HMG-CoAS, FASN, ACLY, IL-1*β, and *IL-1R* was analyzed after 24 h and expression of *IL-6 and TNF*α were analyzed on day 6 using iTaq^TM^ Universal SYBR^®^ Green supermix (Bio-Rad, #172-5124). Samples were analyzed following a quantitation method with efficiency correction, and *TFIIB* was used as a housekeeping gene. Primer sequences are available on request.

### Western Blotting

Protein expression of pro-CASP1, CASP1, Pro-IL-1β and IL-1β was estimated using Western blotting. 2 × 10^6^ human monocytes were seeded in a 6 well cell culture plate. The cells were treated with 2 μM T1317 or left untreated for 24 h and lysed for western blotting. Equal amounts of proteins were loaded on 15% SDS-PAGE. Proteins were transferred on a PVDF membrane (GE Healthcare, #10600023) and blocked in 5% milk (w/v) in Tris–buffered saline supplemented with Tween 20 (TBS-T). Membranes were incubated with a primary antibody pro-CASP1 (Cell Signaling, #3866), cleaved CASP1 (Cell Signaling,#4199), pro-IL-1β (Cell Signaling,#12703); IL-1β (Cell Signaling,#83186); Vinculin Antibody (7F9) (Santa Cruz, Sc-73614), in TBS-T overnight at 4°C, followed by washing with TBST and incubation with a secondary antibody (Goat anti-rb IgG-HRP, Santa Cruz, #sc-2004 or Goat anti-mouse IgG-HRP, Santa Cruz, #sc-2005) in TBS-T for 1 h. The blots were washed as described above, developed using Pierce western blotting substrate (Thermo Scientific, #32106) and images were captured using an Amersham Imager 600 (GE Healthcare). The intensity of bands was quantified and normalized using ImageJ software.

### Lactate Assay

Intracellular Lactate was measured by using a colorimetric L-Lactate assay kit according to the manufacturer’s instruction (Abcam, #ab65330). Cells were cultured in a 6 well plate and treated with T1317 or GW3965 for 24 h. On day 6 cells were washed with ice cold PBS and scraped from the plate and lysed with assay buffer. To eliminate endogenous LDH, cell lysate was deproteinized by spinning through a 10 kD Spin column (Abcam, #ab93349). Absorbance was measured with a CLARIOstar Microplate Reader at 570 nm and the level of lactate was calculated.

### Glucose Consumption Assay

To measure glucose consumption, cells were primed as described above. On day 5 cells were washed once and fresh medium was applied. After 24 h glucose concentration was measured in the supernatant with a colorimetric glucose assay kit by abcam (ab65333), following the manufactures instructions. Absorbance was measured with a CLARIOstar Microplate Reader at 570 nm.

### Acetyl-Coenzyme a Assay

Changes in acetyl co-A concentration were assessed in cell lysate using a colorimetric commercially available kit (Sigma, #MAK039) and performed according to manufacturer’s instructions.

### Seahorse Analysis

Twenty five thousand monocytes per well were seeded in a Seahorse XFp cell culture plate and primed as previously described. The oxygen consumption rate (OCR) was evaluated under basal conditions and in response to 2 μM oligomycin, 1.5 μM FCCP, 100 nM rotenone, plus 1 μM antimycin A (all from Sigma-Aldrich). The cells were cultured in XF-medium (XF Base Medium Minimal DMEM; Agilent Technologies) containing 10 mM glucose, 2 mM l-glutamine, and 1 mM sodium pyruvate (all from Sigma-Aldrich). OCR was determined using a Seahorse XFp Analyzer (Agilent), and assays were analyzed with Wave Desktop software (Agilent), as described before (19).

### Intracellular Staining and FACS

On day 6 primed cells were harvested using ice cold PBS containing 5 mM EDTA. Cells were washed with PBS and staining was performed for 30 min on ice with surface markers for CD80-APC (Biolegend, #305220), CD86-VioBright 515 (Miltenyi Biotec, #130-116-165), CD163-APC-Vio770 (Miltenyi Biotec, #130-112-131), CD206 (Biolegend, #321110) or NLRP3 (Miltenyi Biotec, # 130-111-209) following manufacturer’s instructions. Intracellular staining of p-mTOR and HIF1α was performed after 4% formaldehyde fixation. Cells were washed once with permeabilization buffer (Biolegend, #421002) and stained with PE-Cyanine7 anti-human p-mTOR (Ser2448) (eBioscience, #25-9718-41) or PE anti-human HIF1α Antibody (Biolegend, #359704) antibodies for 20 minutes in the dark at room temperature. PE mouse IgG2b κ (Biolegend) and PE-Cyanine7mouse IgG2a κ (eBioscience^TM^) were used as isotype control for intracellular staining. After staining the cells were washed with PBS and FACS analysis was performed using Guava easyCyte (Millipore).

### Chromatin Immunoprecipitation Assays

Primed cells were harvested on day 6 after crosslinking for Chromatin Immunoprecipitation (ChIP) assays using the MAGnify Chromatin Immunoprecipitation System (Invitrogen) according to manufacturer’s instructions. Chromatin of 200,000 cells was immunoprecipitated with 1 μg of antibody raised against H3k4me3 (Diagenode #C15410003) or H3k27ac (Diagenode #C15410196). Rabbit polyclonal IgG (Diagenode, #C15410206) was used as a negative control. Immunoprecipitated DNA was amplified by quantitative RT-PCR using SYBR green. Enrichment of different histone marks was identified using specific primers for *IL-6* and *TNFα* promoters. The IgG control did not yield a signal in the PCR analysis.

### Statistical Analysis

The differences among experimental groups were evaluated with one-way ANOVA and the Mann–Whitney test using Graph Pad Prism for Windows. An unpaired *t-*test was used to compare the mean levels in case there were only two groups to be compared. A *F*-test was run to check homogeneity of variance before performing analysis. The exact number of individual samples and different experiments is mentioned in the Figure legends. Technical replicates were averaged to limit the impact of experimental variations. A *p*-value of <0.05 was considered to be statistically significant.

## Results

### LXR Agonists Induce a Proinflammatory Phenotype in Human Monocytes

**FIGURE 1:**
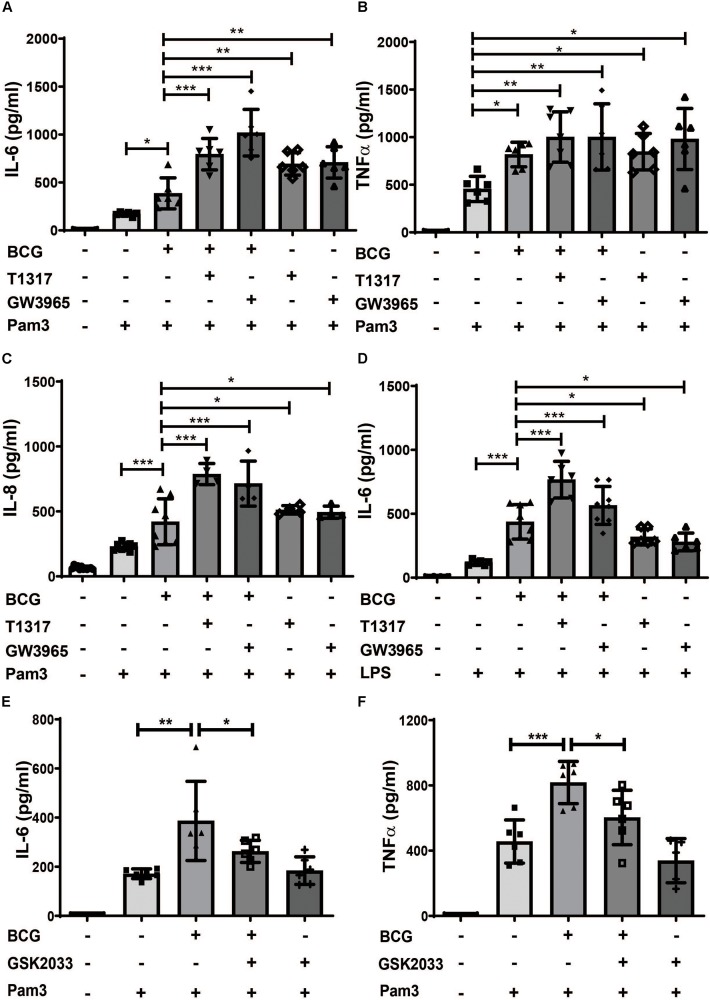
LXR agonists induce a proinflammatory phenotype in human monocytes. Monocytes were treated as indicated with BCG, 2 μM T1317 (LXR agonist), 10 μM GW3965 (LXR agonist), 5 μM GSK2033 (LXR antagonist) or vehicle for 24 h, kept for 5 days in complete medium and restimulated with 5 μg/ml Pam3cys or 10 ng/ml LPS for 24 h. IL-6 **(A,D,E)**, TNFα **(B,F)** and IL-8 **(C)** were measured in the supernatant. Graphs represent mean values ± SD of six individuals in three different experiments. *P < 0.05, **P < 0.01, and ***P < 0.001.

Cholesterol and fatty acid metabolism have been implicated in the regulation of trained innate immunity (10, 14). Since LXR is a master regulator of lipid metabolism we have investigated the role of LXR in BCG-induced trained innate immunity (16). Human monocytes were treated with BCG for 24 h and rested for 5 days to induce macrophage differentiation (20). BCG training induced IL-6, TNFα, IL-8 and MCP-1 synthesis upon restimulation with the TLR2-agonist Pam3cys after 6 days (Figures 1A–C and Supplementary Figure S1A). Pretreatment with LXR agonists (T1317 or GW3965) prior to BCG further increased IL-6 and IL-8 synthesis following Pam3cys restimulation (Figures 1A,C). Restimulation with LPS instead of Pam3cys yielded corresponding results (Figure 1D and Supplementary Figures S1B–D). However, as Pam3cys restimulation provided more consistent data, subsequent experiments were performed with Pam3cys. Next, we pretreated the cells with the LXR antagonist GSK2033 prior to BCG training. As depicted in Figures 1E,F LXR inhibition blocked BCG training. NOD2 activation which is necessary for BCG-induced trained immunity has been shown to induce *LXR*α expression (6, 21). As illustrated in Supplementary Figure S2A BCG treatment also induced *LXR*α expression. Furthermore, LXR inhibition blocked BCG-induced metabolic changes including increased glucose consumption as well as expression of enzymes necessary for increased aerobic glycolysis (Supplementary Figures S2B–E). These experiments demonstrate the relevance of LXR-signaling for BCG-training. However, our most surprising finding was the observation that LXR agonists were able to induce a strong training effect independent of BCG with increased cytokine synthesis upon restimulation with Pam3cys after 6 days (Figures 1A–D for ELISA and Supplementary Figures S1D,E for mRNA). Without restimulation with Pam3cys IL-6 and TNFα levels were not detectable following LXR agonist priming (data not shown).

### LXR Priming Induces an Epigenetic Trained Immunity Phenotype in Human Monocytes

**FIGURE 2:**
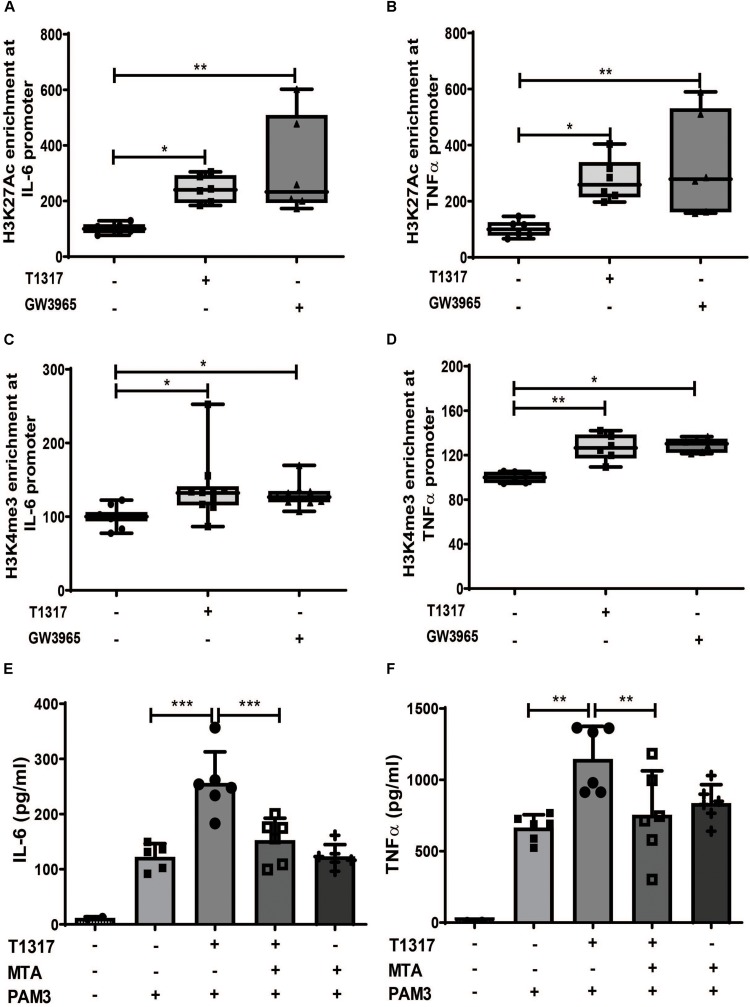
LXR priming induces epigenetic reprogramming. **(A–D)** Monocytes were treated as indicated with 2 μM T1317 (LXR agonist), 10 μM GW3965 (LXR agonist) or vehicle for 24 h and kept for 5 days in complete medium. Chromatin was collected and ChIP assay was performed using an antibody against histone H3 acetylated at lysine 27 (H3K27ac), histone H3 trimethylated at lysine 3 (H3K4me3) or control IgG. Then quantitative real-time PCR was performed with primers for *IL-6* **(A,C)** and *TNF*α **(B,D)**. **(E,F)** Monocytes were treated as indicated with 2 μM T1317 (LXR agonist), 20 μM MTA (histone methyltransferase inhibitor) or vehicle for 24 h, kept for 5 days in complete medium and restimulated with 5 μg/ml Pam3cys for 24 h. IL-6 **(E)** and TNFα **(F)** were measured in the supernatant. Graphs represent mean values ± SD of six individuals in three different experiments. *P < 0.05, **P < 0.01, and ***P < 0.001.

Long-term proinflammatory reprogramming during trained innate immunity is based on sustained activating epigenetic changes on the promoters of inflammatory genes, such as acetylation of H3 lysine 27 (H3K27ac) or histone H3 lysine 4 trimethylation (H3K4me3) (10, 12, 20, 22). Therefore, we investigated whether LXR agonist treatment induces similar changes in human monocytes. As demonstrated in Figures 2A–D priming with LXR agonists resulted in a significant increase of the activating histone modifications H3K27ac and H3Kme3 on the *IL-6* and *TNF*α promoter. Conversely, inhibition of histone methylation with the histone methyltransferase inhibitor MTA has previously been shown to block training with BCG, oxLDL or β-glucan (10). Accordingly, MTA treatment was also able to block LXR training, further asserting the role of epigenetic modifications for the observed phenotype (Figures 2E,F and Supplementary Figures S3A,B). Previous studies have demonstrated that training of monocytes does not induce classical M1 or M2 macrophage phenotypes (9, 23). Similarly, LXR agonist training, although inducing a proinflammatory phenotype, did not induce M1 markers. However, we observed a significant reduction in M2 markers (Supplementary Figures S4A–D).

### LXR Priming Induces a Metabolic Trained Immunity Phenotype in Human Monocytes

**FIGURE 3:**
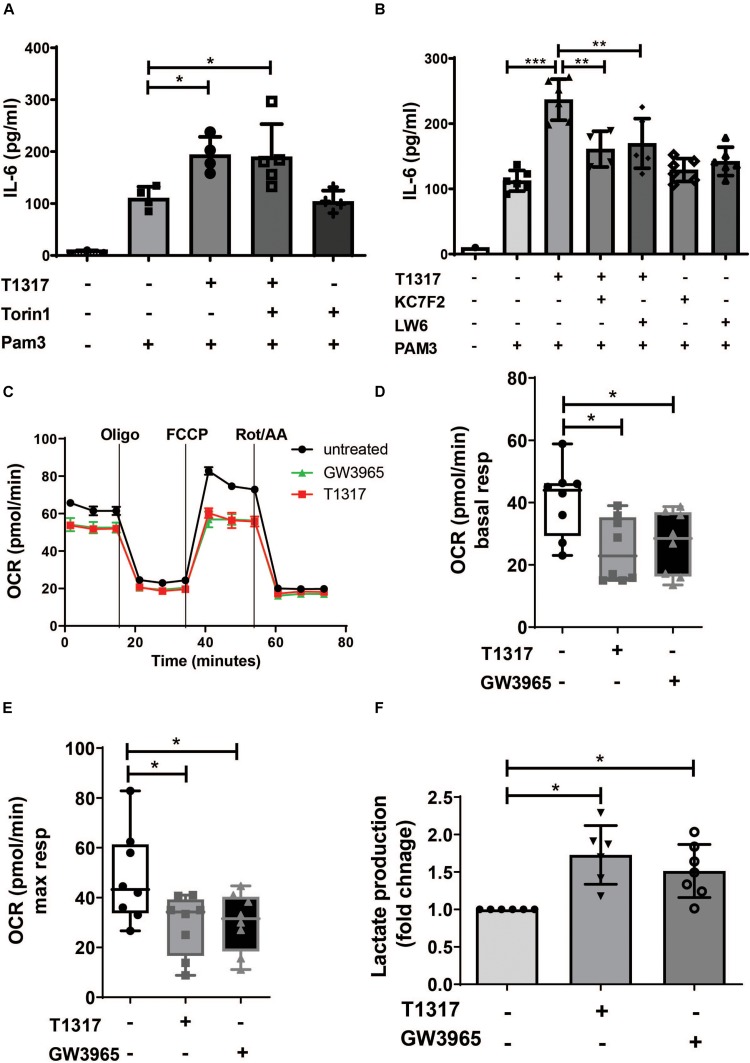
LXR priming induces metabolic reprogramming in trained human monocytes. Monocytes were treated as indicated with 2 μM T1317 (LXR agonist), 10 μM GW3965 (LXR agonist), 100 nM Torin1 (mTOR inhibitor), 10 μM LW6 (HIF1α-Inhibitor), 10 μM KC7F2 (HIF1α-Inhibitor) or vehicle for 24 h and kept for 5 days in complete medium. **(A,B)** Cells were restimulated with 5 μg/ml Pam3cys for 24 h and IL-6 was measured in the supernatant. **(C)** Oxygen consumption rate (OCR) of trained cells was determined on day 6. **(D,E)** Graphs depict mean basal **(D)** and maximal respiration **(E)**. **(F)** On day 6 cells were lysed and lactate concentration was measured. Graphs represent mean values ± SD of six individuals in three different experiments. *P < 0.05, **P < 0.01, and ***P < 0.001.

Metabolic reprogramming is an important hallmark of trained innate immunity. Studies on β- glucan-, BCG- and oxLDL-induced trained immunity have demonstrated a crucial role for aerobic glycolysis that is driven through activation of mTOR and HIF1α-signaling (24). Therefore, we analyzed the role of mTOR and HIF1α in response to LXR activation. As illustrated in Supplementary Figure S5A, LXR activation did not induce mTOR phosphorylation. Furthermore, pharmacologic inhibition of mTOR could not block LXR training (Figure 3A and Supplementary Figure S5C). In contrast to mTOR signaling, HIF1α signaling is necessary for LXR training, as LXR activation moderately induced HIF1α levels (Supplementary Figure S5B) and pharmacologic inhibition of HIF1α was sufficient to block the proinflammatory phenotype (Figure 3B and Supplementary Figures S5D–F). To further analyze the metabolic phenotype of LXR agonist priming we applied the Seahorse XF technology. Cheng et al. have demonstrated that β-glucan training induces a classical Warburg effect in monocytes with decreased oxygen consumption rate (reflecting oxidative phosphorylation) and increased lactate production and aerobic glycolysis (24). Similarly to β-glucan training, LXR agonist priming significantly reduced oxygen consumption rate (Figures 3C–E). Furthermore, lactate production was increased, while the lactate:glucose ratio was close to 2, suggesting that these cells use glucose as the major source of lactate (Figure 3F and Supplementary Figure S6A).

### LXR Priming Depends on the Activity of the Mevalonate Pathway

**FIGURE 4:**
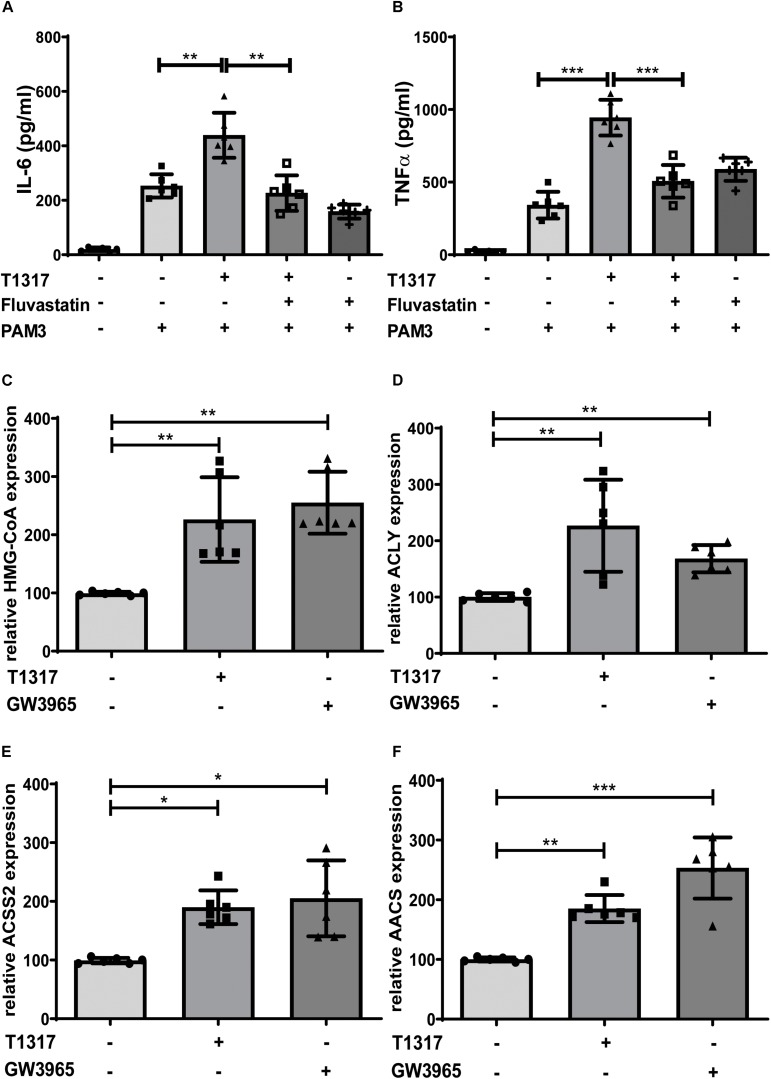
LXR priming depends on the mevalonate pathway. Monocytes were treated as indicated with 2 μM T1317 (LXR agonist), 10 μM GW3965 (LXR agonist), 20 μM Fluvastatin (HMG-CoA reductase inhibitor) or vehicle for 24 h and kept for 5 days in complete medium. **(A,B)** Cells were restimulated with 5 μg/ml Pam3cys for 24 h and IL-6 **(A)** and TNFα **(B)** were measured in the supernatant. **(C–F)** mRNA levels were analyzed by real-time qPCR 24 h after priming. Graphs represent mean values ± SD of six individuals in three different experiments. *P < 0.05, **P < 0.01, and ***P < 0.001.

Bekkering et al. have shown that trained innate immunity depends on the activation of the mevalonate pathway and can be blocked by statins, inhibitors of the rate limiting enzyme of the mevalonate pathway HMG-CoA reductase (10). Similarly, in our experiments, Fluvastatin blocked the LXR induced trained immunity phenotype, demonstrating a similar involvement of the mevalonate pathway (Figures 4A,B and Supplementary Figures S7A,B). Next, we analyzed gene expression profiles of multiple genes involved in cholesterol and fatty acid metabolism in response to LXR activation. Key enzymes of cholesterol and fatty acid synthesis, *HMG-CoA synthase* and Fatty acid synthase (*FASN*), were significantly induced following LXR agonist treatment (Figure 4C and Supplementary Figures S7C). Furthermore, we found several genes involved in the synthesis and metabolism of Acetyl-CoA including *ACLY*, *AACS* and *ACSS2* to be strongly upregulated in response to LXR activation (Figures 4D–F and Supplementary Figures S7D,E).

### LXR Priming Increases Acetyl-CoA Levels, a Novel Mediator of Inflammatory Priming

**FIGURE 5:**
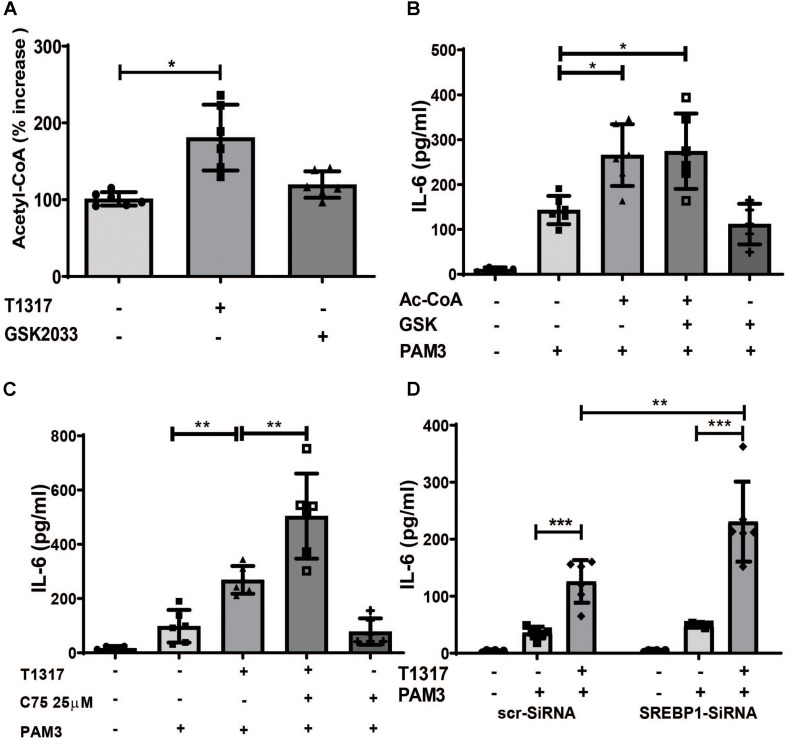
LXR priming increases Acetyl-CoA levels, a novel mediator of inflammatory priming. **(A–C)** Monocytes were treated as indicated with 2 μM T1317 (LXR agonist), 5 μM GSK2033 (LXR antagonist), 500 μM Acetyl-CoA, 25 μM C75 (FASN-inhibitor) or vehicle for 24 h. **(A)** cells were lysed and acetyl-CoA concentration measured on day 3. **(B,C)** cells were kept for 5 days in complete medium and restimulated with 5 μg/ml Pam3cys for 24 h. IL-6 was measured in the supernatant. **(D)** monocytes were transfected with siRNA against *SREBP1* or scrambled siRNA, treated with 2 μM T1317 or vehicle for 24 h and kept for 5 days in complete medium. Then cells were restimulated with 5 μg/ml Pam3cys for 24 h and IL-6 was measured in the supernatant. Graphs represent mean values ± SD of six individuals in three different experiments. *P < 0.05, **P < 0.01, and ***P < 0.001.

Based on the gene expression data, we analyzed the effect of LXR activation on acetyl-CoA levels. As illustrated in Figure 5A, LXR agonist treatment strongly induced Acetyl-CoA levels. Next we investigated a potential role for Acetyl-CoA in trained immunity. Surprisingly, priming of monocytes with Acetyl-CoA was sufficient to induce a proinflammatory phenotype (Figure 5B). In contrast to BCG-priming, Acetyl-CoA priming could not be blocked by LXR inhibition (Figure 5B). Therefore, the mechanism through which Acetyl-CoA induces a proinflammatory phenotype appears to be downstream of the LXR effect. Acetyl-CoA participates in mevalonate pathway as well as the synthesis of fatty acids and Acetyl-CoA accumulation following LXR training could potentially activate both pathways. Since we have already shown, that inhibition of the mevalonate pathway with Fluvastatin blocked LXR training, we then investigated the effect of inhibition of fatty acid synthesis. In contrast to statin treatment, inhibition of fatty acid synthesis using the FASN inhibitor C75 as well as siRNA mediated knock down of *SREBP1*, a key regulator of fatty acid synthesis, superinduced LXR training (Figures 5C,D and Supplementary Figures S8A–E), suggesting an anti-inflammatory role for fatty acid synthesis in this context.

### LXR Training Is Dependent on IL-1β

**FIGURE 6:**
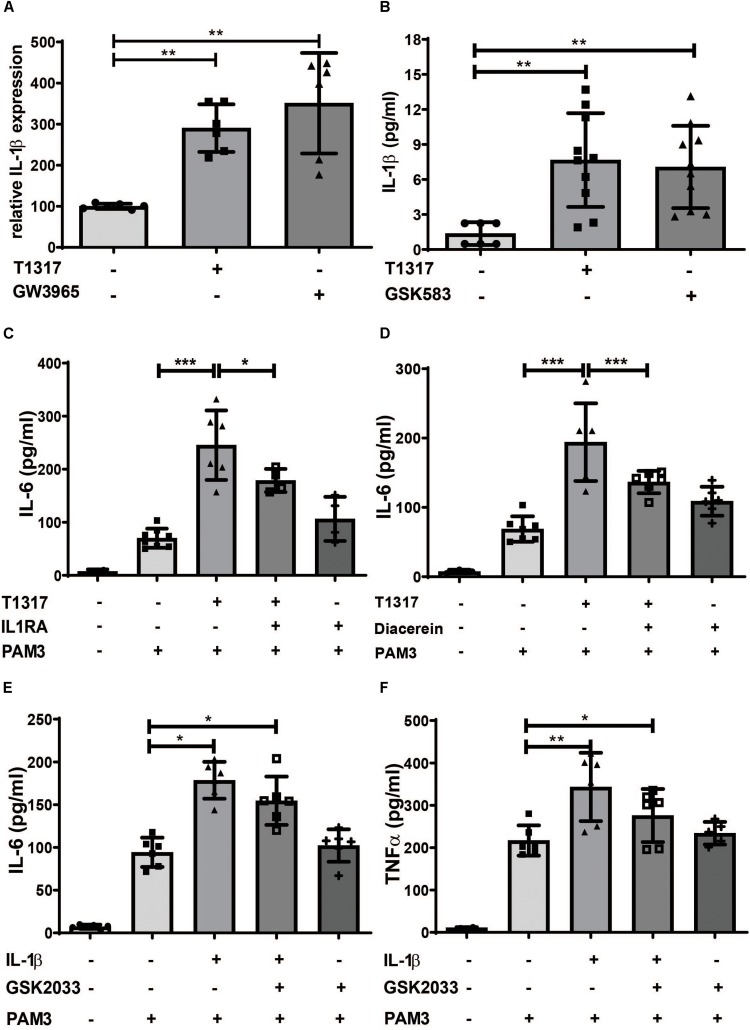
LXR priming is dependent on IL-1β. Monocytes were treated as indicated with 2 μM T1317 (LXR agonist), 10 μM GW3965 (LXR agonist), 5 μM GSK2033 (LXR antagonist), 1 μM Diacerein, 10 ng/ml IL-1β, 200 ng/ml IL1RA (IL-1 receptor antagonist) or vehicle for 24 h. **(A)** mRNA levels of *IL-1*β, were analyzed by real-time qPCR. **(B)** 24 h after priming medium was changed and after 24 h resting time supernatant was collected and the level of IL-1β was measured using ELISA. **(C–F)** cells were kept for 5 days in complete medium and restimulated with 5 μg/ml Pam3cys for 24 h. IL-6 **(C–E)** and TNFα **(F)** were measured in the supernatant. Graphs represent mean values ± SD of six individuals in three different experiments. *P < 0.05, **P < 0.01 and ***P < 0.001.

Another crucial regulator of trained innate immunity is IL-1β. IL-1β is not only necessary in β- glucan-, BCG- and oxLDL-induced trained immunity, but can also directly induce a trained immunity phenotype (11, 14, 25). When we analyzed IL-1β levels in response to LXR activation and Acetyl-CoA priming, we found a strong induction of *IL-1*β and *IL-1R* expression in response to both treatments (Figures 6A,B and Supplementary Figures S9A,D,F–H). Inflammasome activation is a prerequisite for cleavage of pro-IL-1β and secretion of IL-1β and is dependent on caspase-1 and NLRP3. As shown in Supplementary Figures S9B–E LXR agonists induced the expression of caspase-1 and NLRP3, thereby demonstrating inflammasome activation. Inhibition of IL-1β through IL-1β receptor antagonist or diacerein was able to inhibit LXR training (Figures 6C,D and Supplementary Figures S10A,B). Furthermore, priming of cells through IL-1β treatment could not be blocked with a LXR antagonist, indicating a role for LXR activation upstream of IL-1β (Figures 6E,F).

## Discussion

Data demonstrating the fundamental regulation of immune cell functions through metabolic pathways is accumulating rapidly. The modulation of lipid metabolism appears to be of particular importance for short term immune cell activation as well as long-term trained innate immunity. LXR is a key regulator of lipid metabolism as well as innate immune functions, making it a predestined regulator of trained immunity. LXRs are ligand activated nuclear receptor transcription factors that bind to DNA as heterodimers with retinoid X receptors. The LXR subfamily consists of two highly similar isoforms, α and β with different tissue expression patterns. LXRα is strongly expressed in liver, intestine, adipose tissue and macrophages while LXRβ is ubiquitously expressed. When cells accumulate excess cholesterol, oxysterols are synthesized which act as endogenous LXR agonists that directly bind to the LXR ligand−binding domain. The synthetic agonists T0901317 and GW3965 used in our study are full agonists for both LXRα and LXRβ (15, 16).

The concept of trained innate immunity was postulated by Netea et al. It describes the development of a proinflammatory phenotype in response to different stimuli including PAMPs such as β-glucan or BCG, DAMPs such as oxLDL or metabolites such as mevalonate and fumarate (9, 10, 12, 20, 26–28). Training of innate immune cells generates an innate immune memory through metabolic and epigenetic reprogramming resulting in an increased inflammatory response to restimulation (29). The induction of BCG-induced trained innate immunity is dependent on the activation of the NOD2-pathway (6). Interestingly, Joseph et al. have previously shown that addition of the NOD2 ligand muramyl dipeptide to macrophages induced the expression of LXRα mRNA. This induction of LXRα was necessary for the efficient clearance of intracellular bacteria such as Listeria monocytogenes (21). Similarly to other NOD2-ligands, we could show that BCG treatment also induced LXRα expression, while LXR inhibition blocked BCG trained immunity. Studies on β- glucan-, BCG- and oxLDL-induced trained immunity have demonstrated a crucial role for aerobic glycolysis that is driven through activation of mTOR and HIF1α-signaling (8, 24). Surprisingly, training of human monocytes with synthetic LXR agonists was independent of mTOR activation, as we observed no mTOR phosphorylation, and inhibition of mTOR with the specific mTOR inhibitor Torin1 did not block LXR training. However, LXR training still increased lactate production and was dependent on HIF1α as inhibition of HIF1α blocked LXR training. In contrast to LXR training, BCG induced trained immunity is dependent on mTOR activation (20). However, LXR inhibition was able to block BCG induced metabolic reprogramming with reduced expression of glycolytic enzymes and reduced glucose consumption. Therefore, we hypothesize that mTOR activation represents an early phenomenon in BGC trained immunity, which is bypassed by LXR agonists. Apart from aerobic glycolysis, pathway analysis in trained innate immunity has revealed the upregulation of various enzymes of the cholesterol synthesis pathway in response to β-glucan- or BCG training (10, 14). In fact, pharmacologic inhibition of the rate-limiting enzyme in cholesterol synthesis HMG-CoA-reductase prevented the training phenotype (10). Additional experiments identified the cholesterol pathway metabolite mevalonate as a crucial regulator of trained immunity (10). Surprisingly, we found that Acetyl-CoA, the starting point for cholesterol and therefore mevalonate synthesis, can by itself induce a trained immunity phenotype. Furthermore, LXR activation strongly induces enzymes responsible for Acetyl-CoA synthesis such as ACLY as well as intracellular Acetyl-CoA levels, while priming with Acetyl-CoA could not be blocked through LXR inhibition. Therefore, LXR-dependent induction of Acetyl-CoA and the mevalonate pathway represent a potential mechanism for LXR training. In fact, inhibition of HMG-CoA-reductase with Fluvastatin was sufficient to inhibit LXR-training, demonstrating the importance of the mevalonate pathway for the observed phenotype. Besides cholesterol synthesis, Acetyl-CoA also feeds fatty acid synthesis. Although β-glucan training appears to be independent of fatty acid synthesis, as inhibition of fatty acid synthesis by cerulenin had no effect on β-glucan training (12), other data suggest a relevant role for fatty acids in trained innate immunity. For example, Mitroulis et al. recently showed that fatty acid metabolites are involved in the regulation of trained immunity associated effects on hematopoietic progenitors *in vivo* (14), while van der Heijden et al. demonstrated that Aldosterone induced trained immunity is dependent on epigenetically mediated upregulation of fatty acid synthesis and can be blocked by Cerulenin (22). Our data now demonstrate a novel role for fatty acid synthesis in the context of trained immunity as pharmacologic inhibition of fatty acid synthesis with the FASN-inhibitor C75 or siRNA mediated knock down of the key regulator SREBP1 actually increased the inflammatory response following LXR training. Hence, fatty acid synthesis appears to have an anti-inflammatory role in this setting. A potential mechanism for this observation was recently provided by Oishi et al. They have demonstrated that SREBP1-dependent upregulation of fatty acid synthesis is important for the resolution of proinflammatory TLR4-signaling through uncoupling of NFκB-binding to gene promoters (30). Therefore, it is conceivable that anti-inflammatory fatty acids act as a negative regulator of prolonged inflammatory activation.

Interestingly, both LXR activation and Acetyl-CoA treatment strongly induced IL-1β expression. IL-1β is a crucial factor regulating trained innate immunity phenotypes. Extensive studies have demonstrated that IL-1β is necessary for β- glucan-, BCG- and oxLDL-induced trained immunity in murine hematopoietic stem cells *in vivo* as well as human monocytes *in vitro* (11, 14, 25). In fact, in human monocytes trained innate immunity can be induced through priming with recombinant IL-1β (25). In our studies IL-1β was also important for the emergence of the trained phenotype as inhibition of IL-1β signaling attenuated the response to LXR activation. Furthermore, IL-1β priming could not be blocked by LXR inhibition, suggesting a role for IL-1β downstream of LXR. There have been multiple studies suggesting a link between the mevalonate pathway or cholesterol and IL-1β expression or inflammasome activation (31–35). However, there is currently no established mechanism that could explain the observed induction of IL-1β through LXR agonists or Acetyl-CoA and this should certainly be the focus of future studies.

Apart from demonstrating a novel mechanism of trained innate immunity, our study also highlights the previously often underappreciated interspecies variations of LXR functions in humans and mice. Extensive evidence has linked LXR activity to anti-inflammatory effects in murine macrophages and mouse models of inflammatory disease (15, 16). *In vitro*, LXR agonist pretreatment of murine macrophages inhibits the transcriptional induction of inflammatory cytokines in response to TLR-agonists or TNFα (16, 36). *In vivo*, mice lacking LXRs have shown increased inflammation in several different disease models (16, 36), including atherosclerosis (37). Several different mechanisms have been implicated to explain the anti-inflammatory effects of LXR, including transcriptional *trans*-repression at proinflammatory gene promoters, macrophage phenotypic modulation, altered lipid metabolism and changes in plasma membrane signaling via lipid rafts (16). Based on the strong preclinical data, LXR activation has been proposed as a pharmaceutical target to address atherosclerosis formation. Our data demonstrating a strong and sustained proinflammatory activation through LXR agonist treatment in human monocytes are in line with other preliminary studies in human cells. Fontaine et al. have demonstrated that LXR agonists increase TLR4 levels and subsequent inflammatory response to a lipopolysaccharide challenge (18). A similar observation has been made in human dendritic cells (38). Little is known about the mechanisms underlying these species-specific differences. Possible explanations included the differential regulation of TLR4 or lysophosphatidylcholine acyltransferase 3 (39). Our study now extends these observations and provides novel mechanistic data demonstrating LXR dependent metabolic and epigenetic modulation as key factors in this context. Additional studies are necessary to elucidate the detailed mechanisms of the proinflammatory effect of LXR activation, particularly the regulation of IL-1β. However, our evidence certainly warrants additional caution when it comes to the use of LXR agonists in humans.

## Conclusion

In conclusion, we demonstrate that LXR activation induces a proinflammatory trained immunity phenotype in human monocytes through epigenetic and metabolic reprogramming, involving Acetyl-CoA and IL-1β. Our data reveal important novel aspects of LXR signaling and Acetyl-CoA metabolism in innate immunity and warrant additional studies to elucidate the mechanistic differences between mouse and human LXR signaling.

## Data Availability Statement

The datasets generated for this study are available on request to the corresponding author.

## Ethics Statement

The studies involving human participants were reviewed and approved by the Scientific and Ethics Committee of the University of Münster. The patients/participants provided their written informed consent to participate in this study.

## Author Contributions

YS designed the study, performed the experiments, and contributed in writing of the manuscript. GS, LB, SL, ML, and RG performed the experiments. LK assisted in performing the experiments and helped to design the experiments. FK and JW designed the experiments and helped to write the manuscript. HF designed the study and wrote the manuscript.

## Conflict of Interest

The authors declare that the research was conducted in the absence of any commercial or financial relationships that could be construed as a potential conflict of interest.
