# Supplementary material for: LXR Activation Induces a Proinflammatory Trained Innate Immunity-Phenotype in Human Monocytes
Source: Front Immunol. 2020 Mar 10;11:353. doi: 10.3389/fimmu.2020.00353 (PMC7077358; doi:10.3389/fimmu.2020.00353)
Supplement: Supplementary file 1 [file Data_Sheet_1.PDF]

Supplementary Material

LXR Activation Induces a Proinflammatory Trained Innate Immunity-Phenotype in Human Monocytes

Supplemental Figure 1:

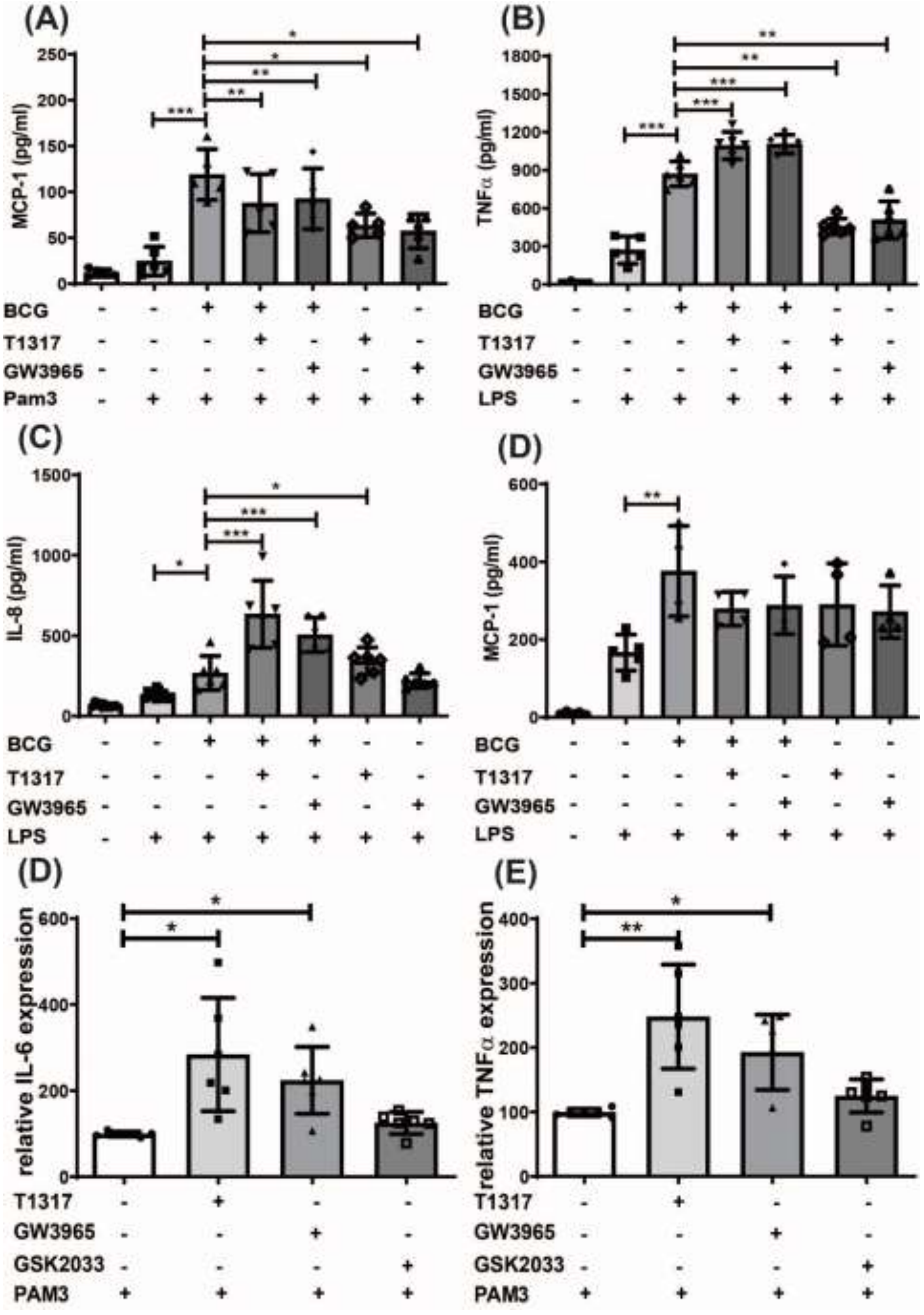

LXR agonists induce a proinflammatory phenotype in human monocytes. Monocytes were treated as indicated with BCG, 2µM T1317 (LXR agonist), 10µM GW3965 (LXR agonist), 5µM GSK2033 (LXR antagonist) or vehicle for 24h, kept for 5 days in complete medium and restimulated with 5 µg/ml Pam3cys for 6h (mRNA)/ with 5 µg/ml Pam3cys or 10 ng/ml LPS for 24h (ELISA). MCP1 (A and D) and TNFα (B) and IL8 (C) were measured in the supernatant. mRNA levels were analyzed by real-time qPCR (D and E). Graphs represent mean values ± SD of 6 individuals in 3 different experiments. *, $P < 0.05$, **, $P < 0.01$ and ***, $P < 0.001$.

Supplemental Figure 2:

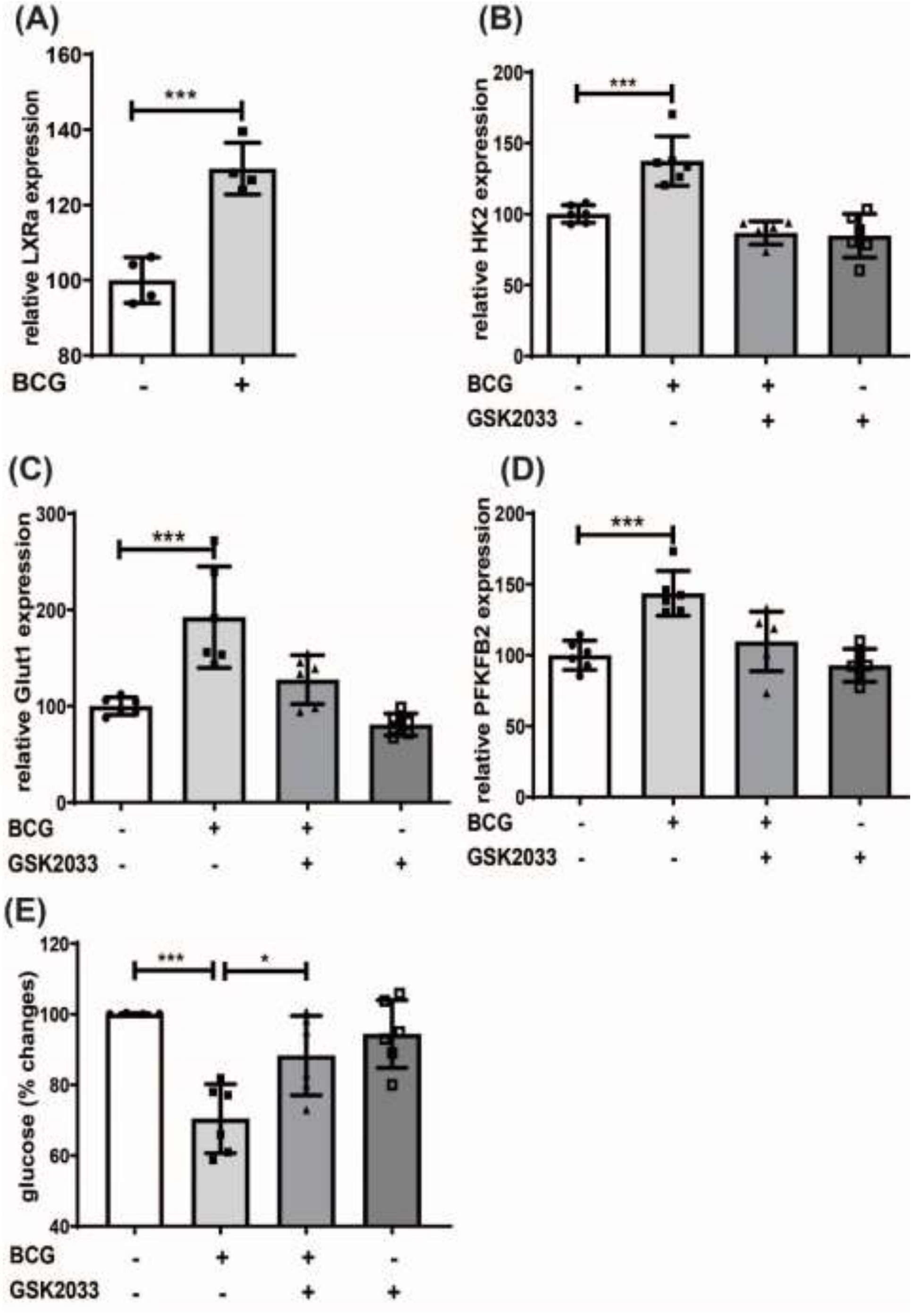

LXR is necessary for BCG-induced metabolic reprogramming. Monocytes were treated as indicated with BCG, 5µM GSK2033 (LXR antagonist) or vehicle for 24h. (A) *LXRa* mRNA expression was analyzed by real-time qPCR 24h after BCG treatment. *HK2* (B), *Glut1* (C) and *PFKFB2* (D) mRNA expression were analyzed by real-time qPCR after 5 days in complete medium. (E) Glucose concentration was measured in medium on day 5 after priming. Graphs represent mean values ± SD of 6 individuals in 3 different experiments. *, $P < 0.05$, **, $P < 0.01$ and ***, $P < 0.001$.

Supplemental Figure 3:

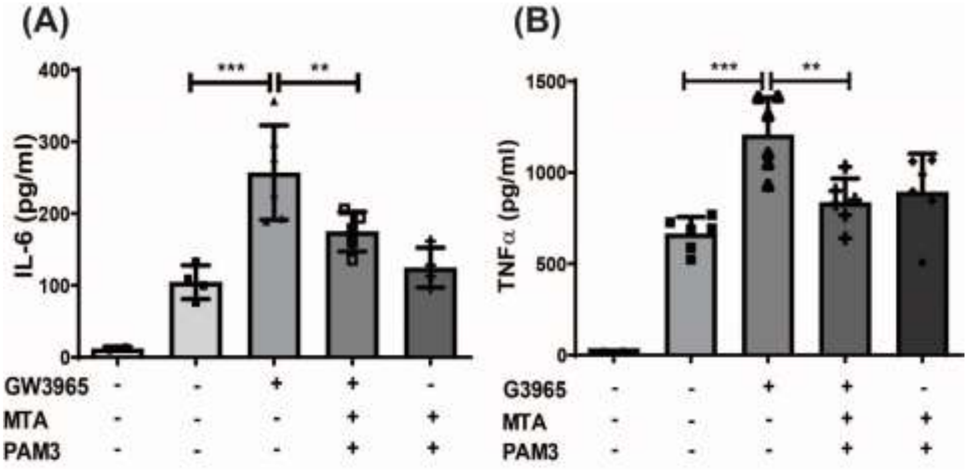

Monocytes were treated as indicated with 10µM GW3965 (LXR agonist), 20µM MTA (histone methyltransferase inhibitor) or vehicle for 24h, kept for 5 days in complete medium and restimulated with 5 µg/ml Pam3cys for 24h. IL6 (A) and TNFα (B) were measured in the supernatant. Graphs represent mean values ± SD of 6 individuals in 3 different experiments. **, $P < 0.01$ and ***, $P < 0.001$.

Supplemental Figure 4:

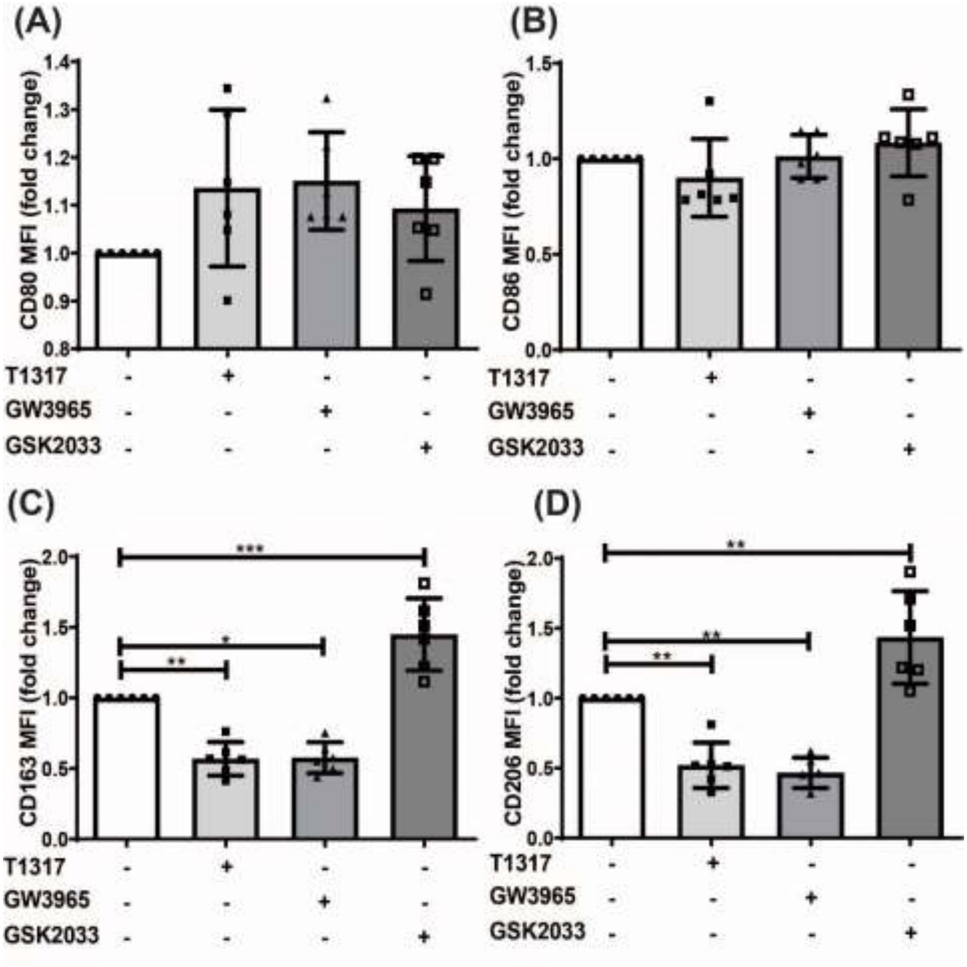

Monocytes were treated as indicated with 2µM T1317 (LXR agonist), 10µM GW3965 (LXR agonist), 5µM GSK2033 (LXR antagonist) or vehicle for 24h and kept for 5 days in complete medium. On day 6 cells were harvested using ice cold PBS containing 5mM EDTA. Cells were washed with PBS, stained with surface markers for CD80 (A), CD86 (B), CD163 (C) or CD206 (D) and analyzed by FACS. Graphs represent mean values ± SD of 6 individuals in 2 different experiments. *, $P < 0.05$, **, $P < 0.01$ and ***, $P < 0.001$.

Supplemental Figure 5:

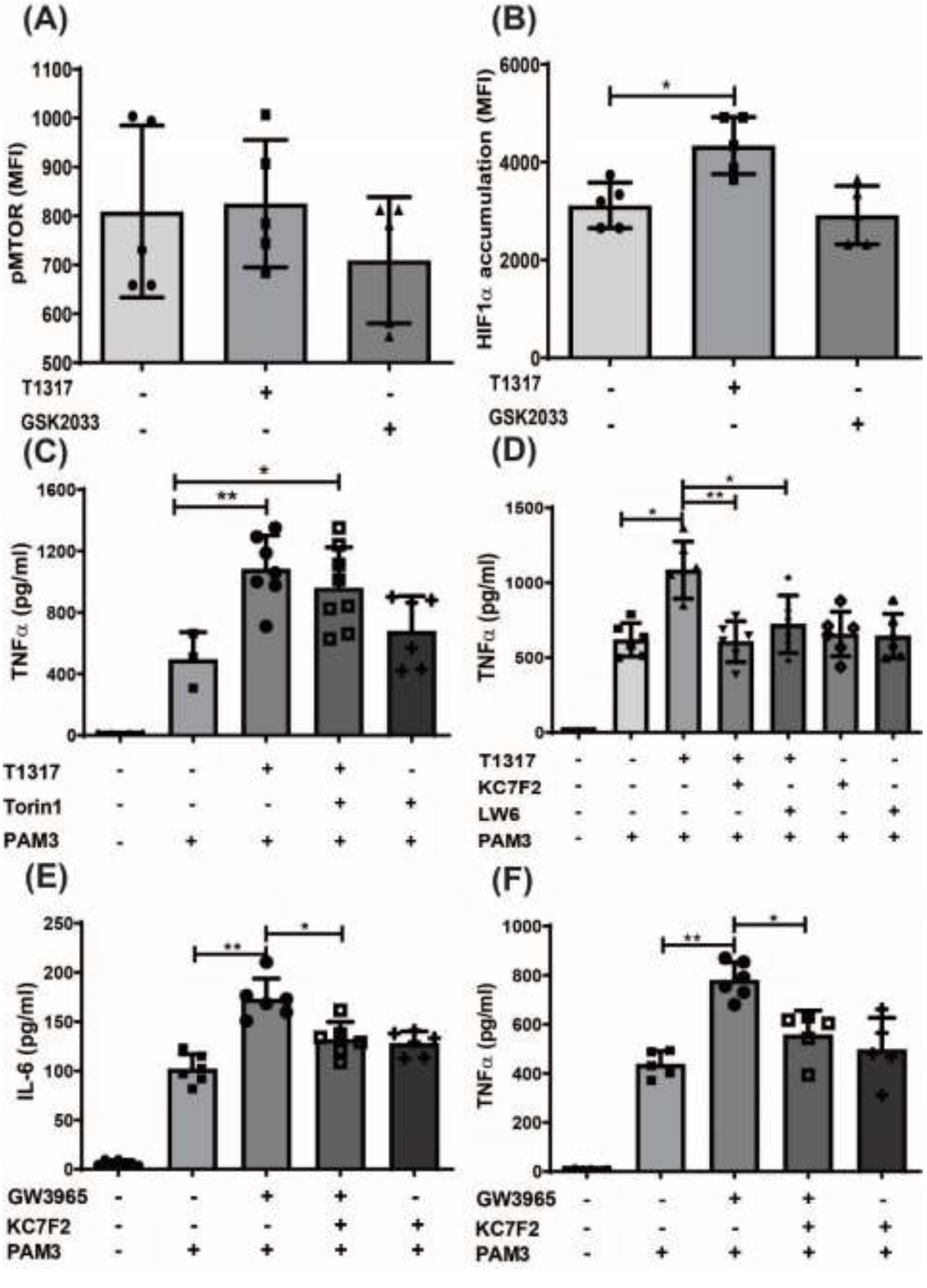

Monocytes were treated as indicated with 2µM T1317 (LXR agonist), 10µM GW3965 (LXR agonist), 5µM GSK2033 (LXR antagonist), 100nm Torin1, 10µM LW6 (HIF1α-Inhibitor), 10µM KC7F2 (HIF1α-Inhibitor) or vehicle for 24h. A and B: Cells were stained with PE-Cyanine7 anti-human p-mTOR (Ser2448) and PE anti-human HIF1α Antibody and analyzed by FACS. C-F: Cells were kept in complete medium for 5 days and restimulated with 5 µg/ml Pam3cys for 24h. IL6 and TNFα were measured in the supernatant. Graphs represent mean values ± SD of 6 individuals in 3 different experiments. *, *P* < 0.05 and ***, *P* < 0.01.

Supplemental Figure 6:

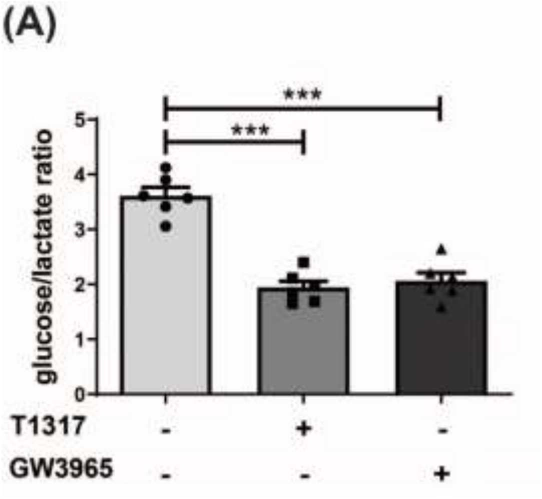

Monocytes were treated as indicated with 2µM T1317 (LXR agonist), 10µM GW3965 (LXR agonist), or vehicle for 24h and were kept in complete medium for 5 days. Medium was refreshed on day 6 and glucose consumption in medium and lactate concentration in cell lysate were measured using commercially available kits. Figure is showing ratio of glucose to lactate concentration. Graphs represent mean values ± SD of 6 individuals in 3 different experiments. ***, $P < 0.001$.

Supplemental Figure 7:

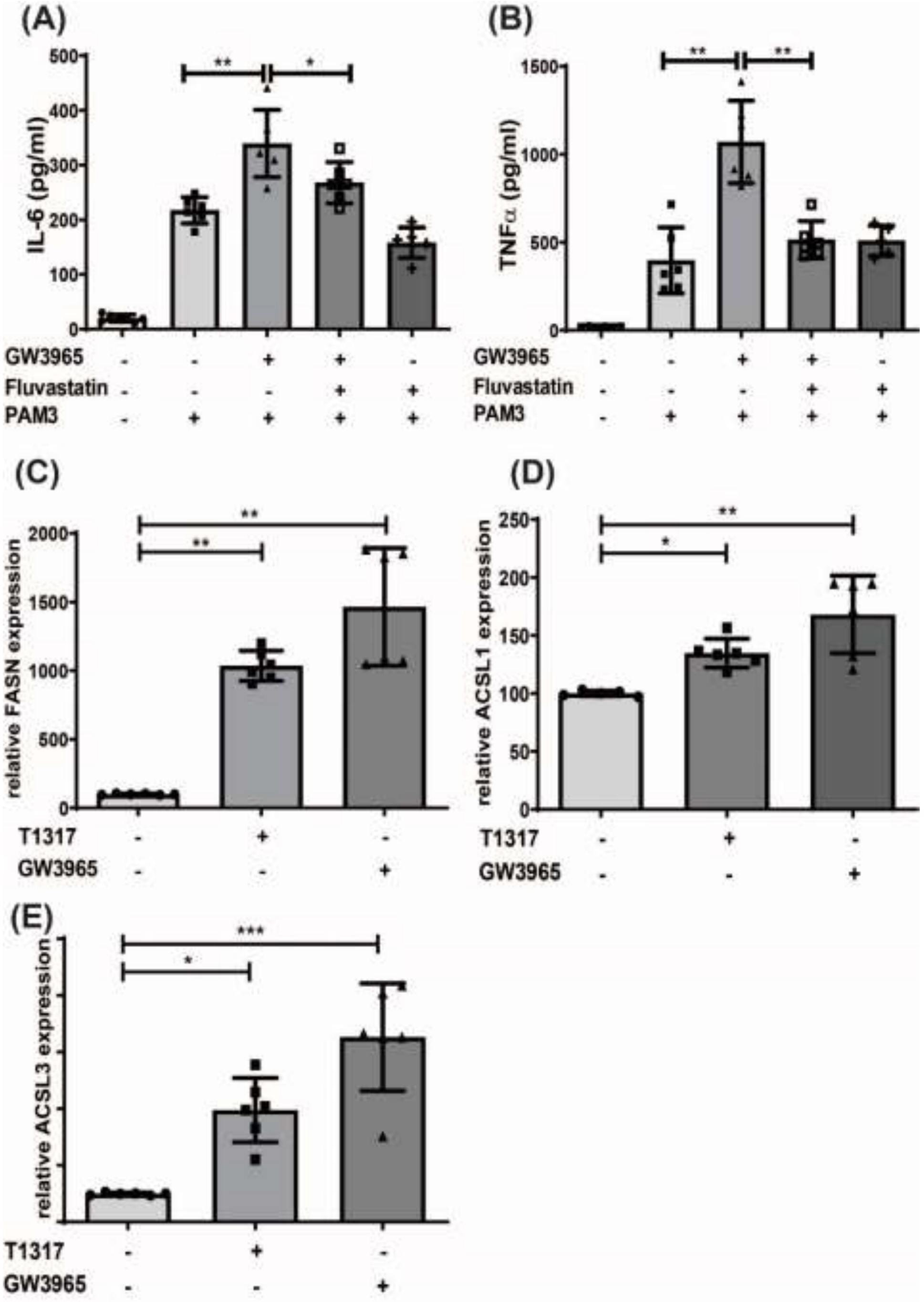

Monocytes were treated as indicated with 2µM T1317 (LXR agonist), 10µM GW3965 (LXR agonist), 20µM Fluvastatin (HMG-CoA reductase inhibitor) or vehicle for 24h. A and B: cells were kept for 5 days in complete medium and restimulated with 5 µg/ml Pam3cys for 24h. IL6 (A) and TNFα (B) were measured in the supernatant. C to E: mRNA levels were analyzed by real-time qPCR 24h after priming. Graphs represent mean values ± SD of 6 individuals in 3 different experiments. *, $P < 0.05$, **, $P < 0.01$ and ***, $P < 0.001$.

Supplemental Figure 8:

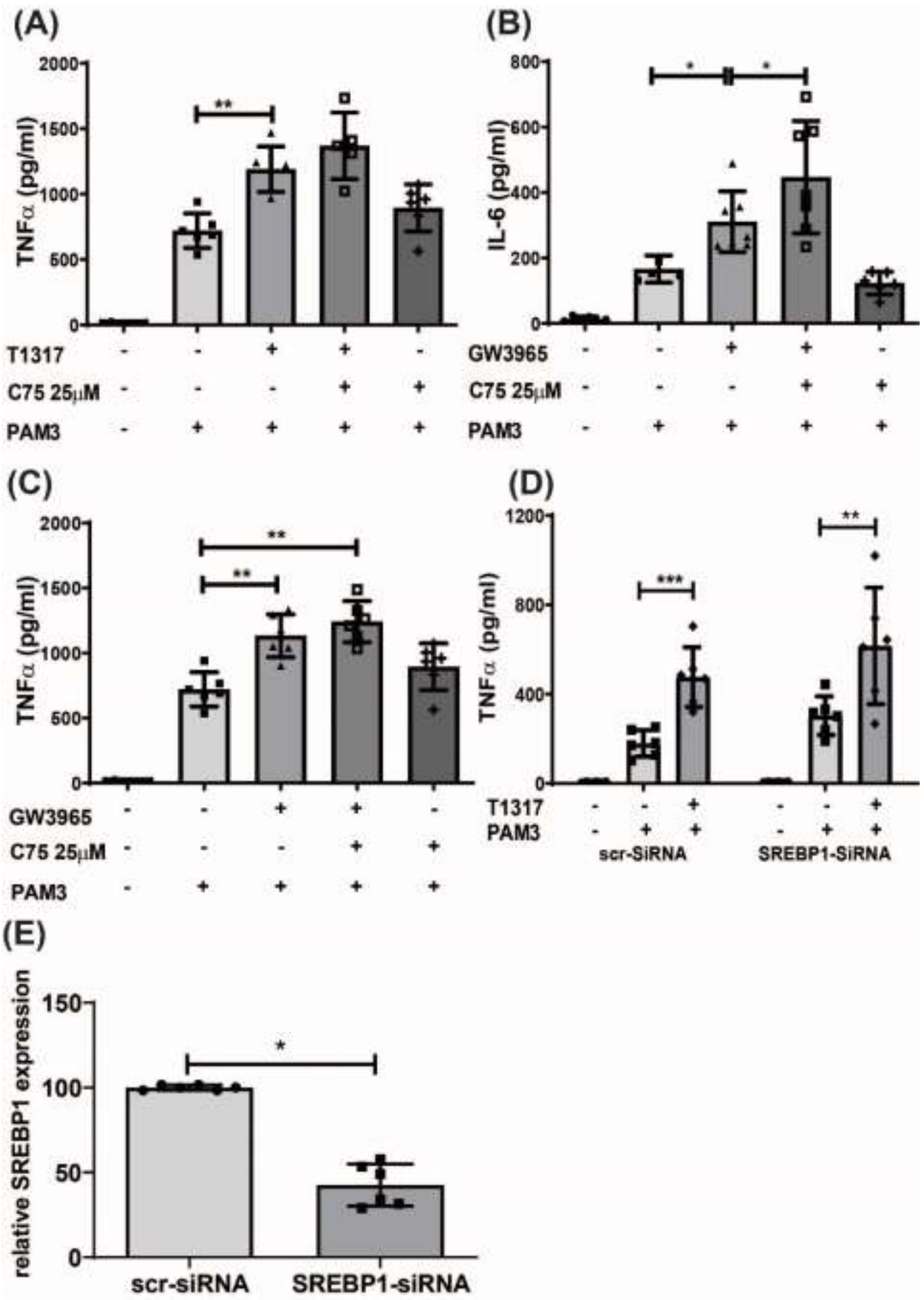

Monocytes were treated as indicated with 2µM T1317 (LXR agonist), 10µM GW3965 (LXR agonist), 25µM C75 (FASN-inhibitor) or vehicle for 24h. A-C: cells were kept for 5 days in complete medium and restimulated with 5 µg/ml Pam3cys for 24h. TNFα (A, and C) or IL6 (B) were measured in the supernatant. D: monocytes were transfected with siRNA against SREBP1 or scrambled siRNA, treated with 2µM T1317 or vehicle for 24h and kept for 5 days in complete medium. Then cells were restimulated with 5 µg/ml Pam3cys for 24h and TNFα was measured in the supernatant. E: For knock down efficiency, mRNA expression of SREBP1 was analyzed 24h after transfection by real-time qPCR. Graphs represent mean values ± SD of 6 individuals in 3 different experiments. *, $P < 0.05$, **, $P < 0.01$ and ***, $P < 0.001$.

Supplemental Figure 9:

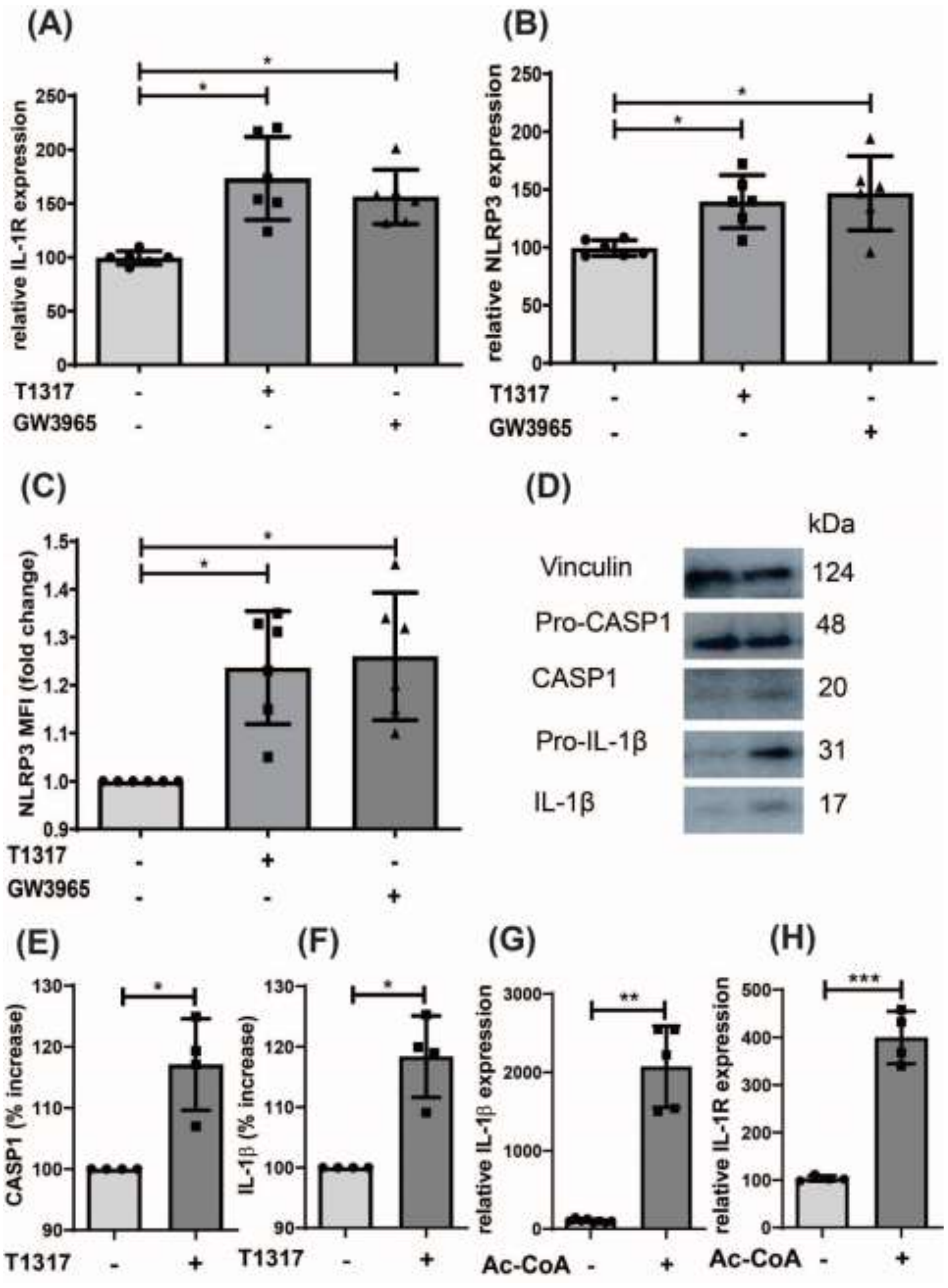

Monocytes were treated as indicated with 2µM T1317 (LXR agonist), 10µM GW3965 (LXR agonist) or vehicle for 24h. A and B: Expression of *IL1R* and *NLRP3* were estimated by real-time qPCR. C: Cells were stained with anti-NLRP3 antibody and analyzed by FACS. D-F: Whole-cell extracts were subjected to western blot analysis for pro-CASP1, CASP1, pro-IL1β and mature IL1β. G and H: Monocytes were treated as indicated with 500µM Acetyl-CoA or vehicle for 24h. mRNA level of IL1β and IL1R were analyzed by real-time qPCR. Graphs represent mean values ± SD of 4 (western blot) or 6 individuals in 2 (western blot) or 3 different experiments. *, $P < 0.05$, **, $P < 0.01$ and ***, $P < 0.001$.

Supplemental Figure 10:

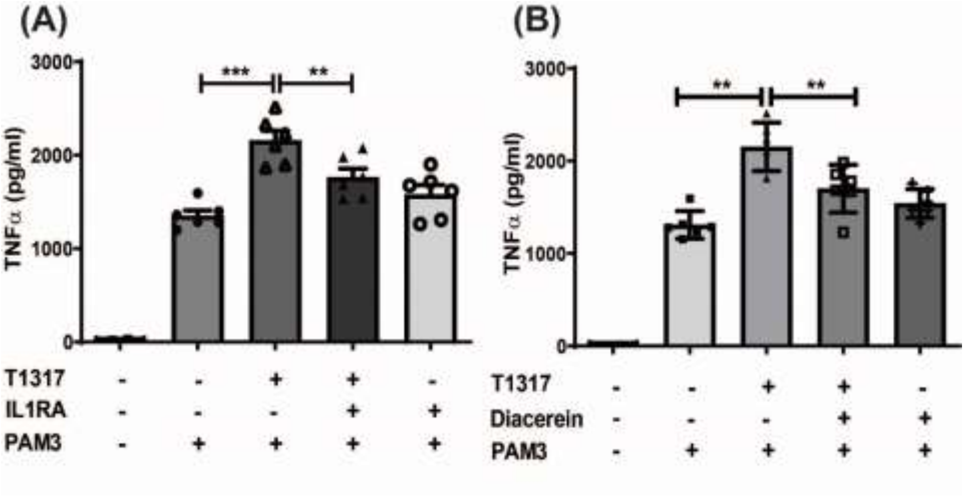

Monocytes were treated as indicated with 2µM T1317 (LXR agonist) 1µM Diacerein, 200ng/ml IL1RA (IL1 receptor antagonist) or vehicle for 24h kept for 5 days in complete medium and restimulated with 5 µg/ml Pam3cys for 24h. TNFα was measured in the supernatant using ELISA. Graphs represent mean values ± SD of 6 individuals in 3 different experiments. *, $P < 0.05$, **, $P < 0.01$ and ***, $P < 0.001$.
